# A rare foreign body migration: from head to neck

**DOI:** 10.1016/j.bjorl.2021.03.012

**Published:** 2021-04-24

**Authors:** Vedat Delibas, Muhammet Rasit Muharremoglu, Kemal Koray Bal, Sedat Alagoz

**Affiliations:** Adana City Training and Research Hospital, Otorhinolaryngology Department, Adana, Turkey

## Introduction

The head and neck region contains many vital anatomical structures. Therefore, diseases and trauma in this region may have a more morbid and mortal course compared to other anatomical regions. The neck can be anatomically delimited by the body of the mandible and the mastoid bone at the top, the trapezius muscle laterally, the clavicula below and an imaginary line passing through the midline of the neck medially. The main arteries of the neck are the common carotid, and the main veins that collect blood from the head and face are the external and internal jugular veins. Penetrating neck trauma accounts for approximately 5–10% of all trauma cases presenting to the emergency department. Approximately 30% of these cases are accompanied by injuries other than in the neck regions. The current mortality rate in patients with penetrating neck injuries ranges from 3% to 6%. In this article, a 2 year-old patient with torticollis from a foreign body in the temporoparietal scalp descending to the sternocleidomastoid muscle in about 2 months from the effect of gravity as a result of head trauma, presented in the light of the literature.

## Case presentation

**Figure 1:**
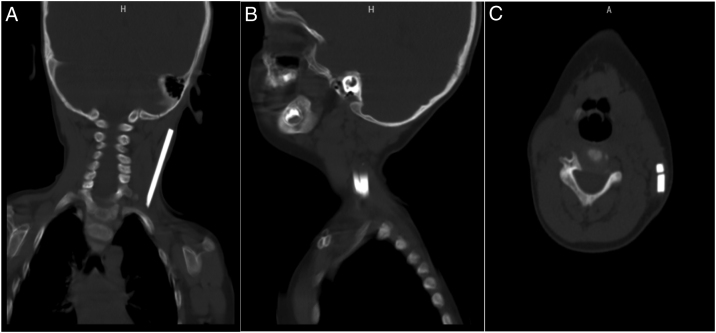
(A) Preoperative foreign body in neck CT images coronal section; (B) Preoperative foreign body in neck CT images sagittal section; (C) Preoperative foreign body in neck CT images axial section.

**Figure 2:**
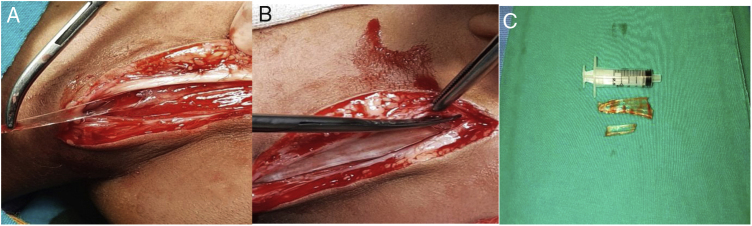
(A) Intraoperative foreign body removal; (B) Intraoperative foreign body is released from surrounding tissue; (C) Foreign bodies removed from the neck (two glass pieces, 7 and 3 cm).

**Figure 3:**
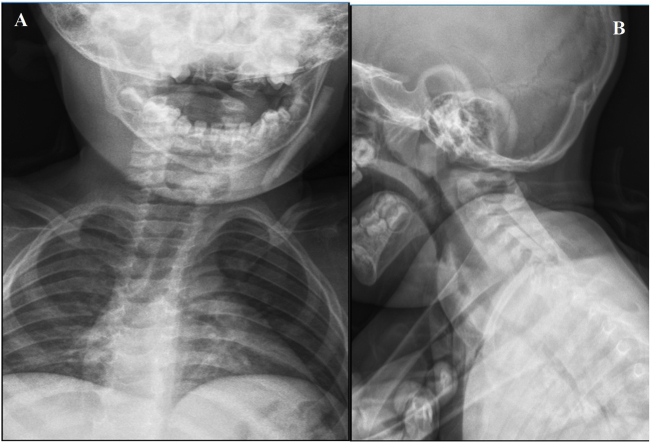
(A) Scope image taken intraoperatively anteroposterior view; (B) scope image taken intraoperatively lateral view.

A 2-year-old boy who was known to be healthy before, was evaluated in the emergency department due to left-sided torticollis. The patient was being followed up in the physical therapy and rehabilitation department of the external hospital for torticollis. The child was restless and agitated on examination while investigating the cause of torticollis. There was limitation in neck movements and pain. There were no areas of hyperemia, warmth and fluctuation on the skin of the neck, and no trismus existed. There was no neck stiffness, there was no fever and the blood picture was normal except for a slight increase in C-reactive protein. Other ear-throat-nose examinations were normal. There was a palpable 3 × 1 cm partially mobile, hard mass in the left side posterior cervical / infraauricular region. There was no incision or scar on the skin of the neck. Nothing was detected in the outside hospital imaging. Focal echogenicity in the left sternocleidomastoid muscle in our ultrasound revealed a tubular foreign body extending along the posterolateral part of the left sternocleidomastoid muscle on neck tomography (Fig. 1A–C). When neck exploration was performed, it was observed that there were two glass pieces, approximately one 7 cm and the other 3.5 cm, on the left sternocleidomastoid muscle (Fig. 2A and B). The foreign bodies were carefully removed (Fig. 2C) and the wound was irrigated with 10% betadine. The operation was terminated after confirming that there were no another foreign bodies with intraoperative fluoroscopy, and suture/closure of the wound was applied (Fig. 3A and B). When the patient's family was questioned later, it was learned that a glass cabinet had fallen on him 4 months ago and an approximately 5 × 2 cm wound was formed in the scalp on the left temporoparietal region on his head, and that the emergency department and suture/repaired the wound. In the postoperative period, the patient's torticollis was resolved and there was no complication in the 3 month followup.

## Discussion

Head and neck traumas can be fatal, especially in children, as the head contains vital structures. Isolated neck penetrating trauma is uncommon and rarely serious in children. Approximately 50% of neck injuries in children are penetrating and the other half is blunt.^1^

Foreign bodies in the soft tissue of the neck often develop as a result of penetrating trauma, and these foreign bodies often fail to be removed or become stuck in the area where they enter, or in a very rare probability, the foreign body taken from the aerodigestive tract has a sharp surface like a fishbone and can migrate to the neck soft tissue by creating its own paths.^2^, ^3^ In our case, the glass fragments entering from the scalp in a very rare way were detected on the sternocleidomastoid without any incision or scar marks on the neck skin. In the case report of Sreetharan et al., It was stated that fish bones in the oropharynx and larynx can migrate to thyroid nodules, neck soft tissues and structures in the neck.^2^ Apart from these presentations, iatrogenic reasons can also cause foreign bodies in the neck.^4^, ^5^ In the case report of Karakida et al., It was found that the local anesthetic needle used during the dental procedure was broken in the molar gingiva and then appeared in the right subcutaneous tissue of the neck.^4^ Altay et al, in their case report, presented the migration of the broken dental needle to the post-cervical region in the inner surface of the mandible.^5^ Interesting cases missed after traffic accidents are also available in the literature. Sreetharan et al., reported an ingested tooth after a traffic accident, seen in the soft tissues of the neck near the right lobe of the thyroid.^6^ Ahmad et al. observed that foreign bodies (wood pieces) remained in the neck of a patient who had a penetrating injury on the right side of the neck after a traffic accident which was closed primarily after the first intervention and then a retro/parapharyngeal abscess formed.^7^ In the case report of Ko and Lee, the k-wire they used for the right acromioclavicular joint was observed in the left side soft tissue of the neck after migration.^8^ In our case, foreign bodies was not taken orally and migrated down the scalp. It was in the form of foreign bodies that entered the scalp incision after head trauma to migrate to the post-cervical area of the neck, without damaging any nerves or vital vessels. A similar situation has not been found in the literature since the glass pieces give the image of a mass in the neck area.

Foreign bodies in the soft tissue of the neck can cause nerve and vascular injury with a sharp surface, complications in general, nerve injuries such as retro/parapharyngeal abscesses, deep neck infections, aorto-esophageal fistulae, mediastinitis and tracheo-esophageal fistula, subcutaneous emphysema, pneumomediastinum, ans facial and laryngeal nerve injury. Our case was also a unique case because it caused torticollis in a way that has not been described before.

The visualization methods may vary according to the nature of the foreign body. In the acute period in wood penetrating injuries, a foreign body remaining inside may be missed. CT is superior to non-metallic foreign bodies compared to magnetic resonance.^9^

In general, while preparation for foreign bodies in the neck is made in the preoperative phase, there are some methods that will guide intraoperation. Some authorities advocate that high resolution USG is a useful and new approach to detect the location of radiopaque or non-opaque foreign bodies in the intraoperative period to assist in removal of all objects, especially in cases with multiple foreign bodies.^10^ We determined the localization of foreign body with CT in the preoperative period. In the intraoperative period, we confirmed that there was no residual foreign body left with fluoroscopy.

## Conclusion

Head traumas are important in children and may require clinical followup and imaging. The inside of the potential cavity that occurs in the lacerations located on the scalp should be thoroughly washed with saline and after the bleeding is controlled, it should be sutured after making sure that there is no foreign body inside. Otherwise, they may cause complications requiring surgery to avoid potential fatal complications involving the circulatory and respiratory system.

## Conflicts of interest

The authors declare no conflicts of interest.
